# Comparison of Promeristem Structure and Ontogeny of Procambium in Primary Roots of *Zea mays* ssp. *Mexicana* and *Z. mays* ‘Honey Bantam’ with Emphasis on Metaxylem Vessel Histogenesis

**DOI:** 10.3390/plants8060162

**Published:** 2019-06-08

**Authors:** Susumu Saito, Teruo Niki, Daniel K. Gladish

**Affiliations:** 1 Department of Biotechnology, Takushoku University, Tatemachi 815-1, Hachioji, Tokyo 193-0985, Japan; mdjmk210@ybb.ne.jp (S.S.); tniki@la.takushoku-u.ac.jp (T.N.); 2 Biological Sciences Department, Miami University, 1601 University Blvd, Hamilton, OH 45011, USA

**Keywords:** metaxylem, root development, procambium, histogen, *Zea mays*, teosinte

## Abstract

Classical histology describes the histological organization in *Zea mays* as having a “closed organization” that differs from *Arabidopsis* with the development of xylem conforming to predictable rules. We speculated that root apical meristem organization in a wild subspecies of *Z. mays* (a teosinte) would differ from a domestic sweetcorn cultivar (‘Honey Bantam’). Careful comparison could contribute to understanding how evolutionary processes and the domestication of maize have affected root development. Root tips of seedlings were prepared and sectioned for light microscopy. Most sections were treated with RNase before staining to increase contrast between the walls and cytoplasm. Longitudinal and serial transverse sections were analyzed using computer imaging to determine the position and timing of key xylem developmental events. Metaxylem development in *mexicana* teosinte differed from sweetcorn only in that the numbers of late-maturing metaxylem vessels in the latter are typically two-fold greater and the number of cells in the transverse section of procambium were greater in the latter, but parenchymatous cell sizes were not statistically different. Promeristems of both were nearly identical in size and organization, but did not operate quite as previously described. Mitotic activity was rare in the quiescent centers, but occasionally a synchronized pulse of mitoses was observed there. Our reinterpretation of histogen theory and procambium development should be useful for future detailed studies of regulation of development, and perhaps its evolution, in this species.

## 1. Introduction

Maize is believed to have been domesticated beginning about 9000 years ago from one of several species of large grasses, commonly known as “teosinte”, native to what is now central Mexico [1,2]. “Teosinte” is no longer considered to be one species separate from maize (i.e., domesticated *Z. mays*). This common name is now understood to have been traditionally applied by indigenous people to three non-maize species (*Z. perennis*, *Z. diploperennis*, and *Z. luxurians*) and three subspecies of *Z. mays* (ssp. *huehuetenangensis*, *mexicana*, and *parviglumis*), all of which have older natural histories than domesticated cultivars of *Z. mays* and are more similar to each other structurally than they are to domesticated maize. The inclusion of the latter three into species *Z. mays* was done by virtue of the “reproductive criterion” (all these subspecies freely hybridize with domestic maize to produce fertile F1 plants) as well as by morphological, geographical, isozyme, and DNA RFLP analysis criteria [1,2].

A study of the possible influences that domestication of maize from Balsas teosinte (*Z. mays* ssp. *parviglumis*) had on gametophyte and embryo development has been done, and significant shifts in timing and patterning were revealed that account for the larger size of the caryopses of maize [3]. Other aspects of development have been similarly subject to the influence of selection during domestication, for example, increased apical dominance leading to changes in the pattern of branching and the morphology of their infructescences [2]. Several morphological and anatomical parameters of mature root systems were recently shown to differ significantly between wild teosintes and domesticated *Z. mays* subspecies, notably with proportionally larger stele and xylem vessel cross-sectional areas in domesticated maize [4] that possibly contribute to increased yield by increasing vascular capacity. Because we observed that their primary roots are morphologically different (maize primary roots have much larger diameters than teosinte) despite being members of the same species, we were interested in seeing if an anatomical analysis of primary root apical meristem organization among the subspecies of *Z. mays* could similarly contribute to our understanding of how evolutionary processes and domestication might have affected developmental patterns in the primary roots of domesticated maize compared to *Z. mays* teosintes. A study of this kind in roots has only been done for an unusual family of dicotyledons: Podestemaceae, a family that has rather distinctive root morphology [5]. Roots of *Z. mays* are particularly well suited to precise anatomical analysis because their closed apical organization and predictable histogenesis make measurements more manageable and meaningful (e.g., [6]). Furthermore, genetic relationships have been thoroughly assessed among the sub-species of *Z. mays* [2].

Over the last 150 years much work has been invested in evaluating and understanding the structure and patterns of histogenesis in root apical and primary meristem zones (RAMs). Our general understanding of RAM structure and behavior was well reviewed by Jiang and Feldman [7] and Heimsch and Seago [8]. Key events in the histogenesis of xylem tissue were mapped by Feldman [6] in a commercial maize cultivar (*Zea mays* ‘Kelvedon 33′), and he reported that the first four metaxylem files “differentiated simultaneously” and that metaxylem differentiation was correlated with quiescent center size.

The cellular organization of the apical meristems of primary roots of flowering plants has been described as being “closed” or “open” [8,9,10,11,12] depending upon whether mitotically active initials (stem cells) are or are not, respectively, associated at the margins of distinct layers of cells in the promeristem, called histogens [13]. In the closed type there is a distinct boundary, called the root cap junction (RCJ), between the root cap and the other promeristem tissues, and most cell files appear to emerge and proliferate from the distinct histogen layers, while in the open system they do not [7,8,12,14]. Intermediate types have been described, as well as species whose roots change over time [8,9,12,15,16].

The best-characterized example of a closed system is that of *Arabidopsis thaliana*, which is a dicotyledonous plant in the Brassicaceae [16,17,18]. While Dolan et al. [17] gave cursory regard to the accepted nomenclature, Baum and Rost [18] and Baum et al. [16] described in detail the three histogen layers (tiers) during early root development that are small uniseriate disks, occasionally biseriate at their margins, transversely stacked in the following sequence: (1) the dermatogen-calyptrogen complex has central cells that divide periclinally to the histogen layers where they abut the base of the root cap columella to produce additional columella cells. The calyptrogen central cells are surrounded by a collar of peripheral cells that variously divide periclinally and anticlinally to produce the protoderm and peripheral root cap initials that subsequently further proliferate to produce the protoderm and peripheral root cap tissues. (2) Basipetal from the dermatogen-calyptrogen is the periblem, a similar small disk whose central cells rarely divide, but whose peripheral cells variously divide anticlinally and periclinally to obliquely produce the three-layered cortex (ground meristem). The innermost layer of this tissue differentiates into the endodermis. (3) Basipetal from the periblem is a small disk of cells that is called the plerome. These cells divide transversely to the root axis (periclinal to the histogen layer) to produce the tissues of the procambium in a manner akin to the central cells of the dermatogen/calyptrogen, except that proliferation is in the opposite direction. The diarch sectoral histological organization typical of the stele of *A. thaliana* can be detected in the plerome because cells that divide to form cell files that will become tracheary elements are slightly larger than the flanking cells that produce differentiating phloem cell types. The outermost cells of the plerome produce a uniseriate pericycle by anticlinal divisions transverse to the root axis. Some aspects of the molecular genetic regulation of primary root vascular development have recently been revealed in the *Arabidopsis* system [7,19,20,21,22,23]. In *A. thaliana*, which has very narrow roots, four cells occupy the center of the periblem, and these, by virtue of their mitotic inactivity, constitute the quiescent center (QC) [17,24], which is significantly larger in other species in which it has been reported [6,7,24].

Plants in the Poaceae (grasses) typically have root apical meristems with closed organization that also have defined histogen layers, though the organization differs slightly from *Arabidopsis*. In grass root apices the uniseriate root cap-producing calyptrogen is usually quite distinct and the protoderm and ground meristem are produced by a single dermatogen-periblem histogen layer immediately basipetal to it. As in *Arabidopsis*, the procambium that becomes the pericycle, xylem, and phloem of the stele is reportedly produced by the uniseriate plerome layer [25,26]. Careful anatomical and mitotic activity studies relevant to root apical development have been performed mainly on economically important domestic grasses such as wheat (*Triticum*) [14], oats (*Avena sativa*) [27], barley (*Hordeum sativum*) [28,29,30], rice (*Oryza sativa*) [26], and especially maize (*Zea mays*) [6,7,14,29,31,32,33,34]. Some of these authors have specified patterning “rules” and made proposals as to the role that metaxylem element differentiation plays in establishing and maintaining the patterns of development.

Unfortunately, the numerical precision and predictability of the *Arabidopsis* root system is not typical of most plant species. Furthermore, errors of interpretation due to misunderstood terminology or due to oversimplification and/or extrapolation from model systems such as *Arabidopsis* that are uncharacteristically simple by comparison to most plant species can lead to confusion, for example, in the otherwise excellent review by Miyashima et al. [20]. It is important therefore that increasingly precise and careful studies of plant species other than *Arabidopsis thaliana* continue to be undertaken so that as increasingly effective and versatile molecular tools are developed the histological topology of other plant systems shall have been mapped in detail and such new strategies can be used with precision. This has been recently done well for roots of a range of dicotyledonous plants [35]. Here we continue that effort for monocotyledonous plants and endeavor to add insight into the underlying developmental mechanisms that became altered in *Z. mays* during the artificial selection typical of domestication.

The objectives of the present study were (1) to use a new approach to slide preparation that more clearly reveals structural patterns at the tissue and cell level to enhance precision thin-sectioning and high magnification analysis of RAM structure, procambium development, and late-maturing metaxylem vessel initiation in an undomesticated *Z. mays* subspecies (*mexicana*) and a modern, highly-derived commercial cultivar (*Z. mays* ‘Honey Bantam’); and (2) to evaluate the anatomical differences that that may contribute to our understanding of the factors that cause dimensional differences apparent in their primary root tips. Preliminary data led us to believe that some earlier analyses of maize closed root apical organization that relied heavily on longitudinal sections and coarser levels of resolution due to the methods prevalent at the time missed some important details of the development process in this species. This ultimately led us to reassess the way histogens have been defined and the way they behave in *Zea mays* RAMs.

## 2. Results

### 2.1. RNase Treatment before Staining

**Figure 1:**
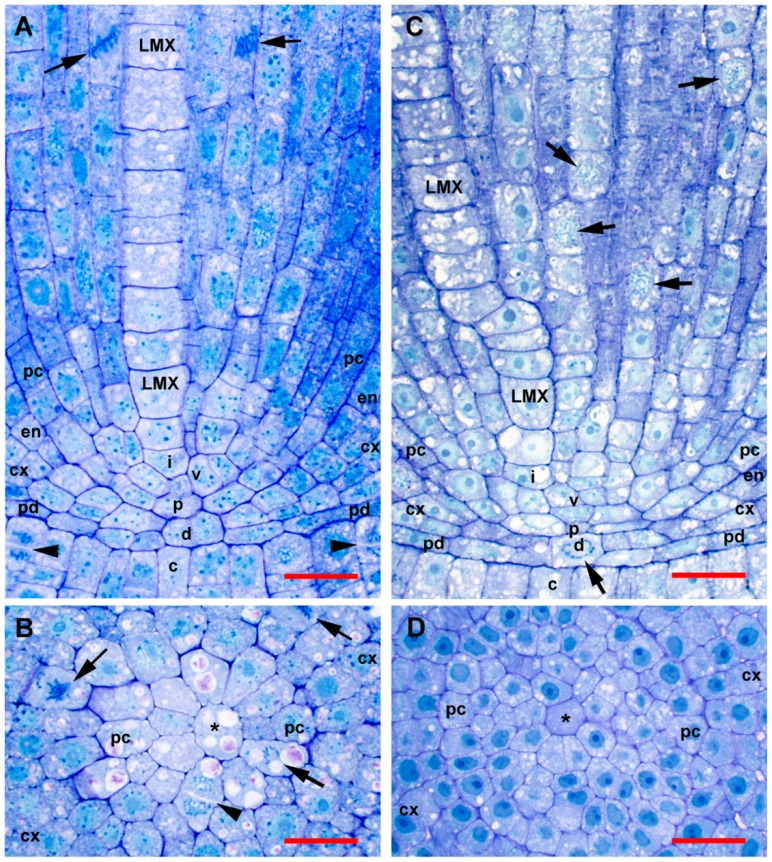
Organization of tissues in the promeristem and primary meristem zones of teosinte and sweetcorn roots at high magnification. Sections were not RNase treated (compare to Figure 2 and Figure 3). (**A**) Slightly off-median longitudinal section of teosinte. (**B**) Transverse section through the plerome of teosinte showing margin initial during cytokinesis. (**C**). Slightly off-median longitudinal section of sweetcorn. (**D**) Transverse section through the plerome of sweetcorn. LMX, late-maturing metaxylem vessel; asterisk (*), plerome central cell; arrow, mitotic cell; arrowhead, newly forming cell plate; c, calyptrogen; cx, immature cortex; d. dermatogen-periblem complex; en, immature endodermis; i, LMX initial cell; p, plerome; pc, pericycle; pd, protoderm; v, vascular initials layer. Scale bar = 25 µm.

**Figure 2:**
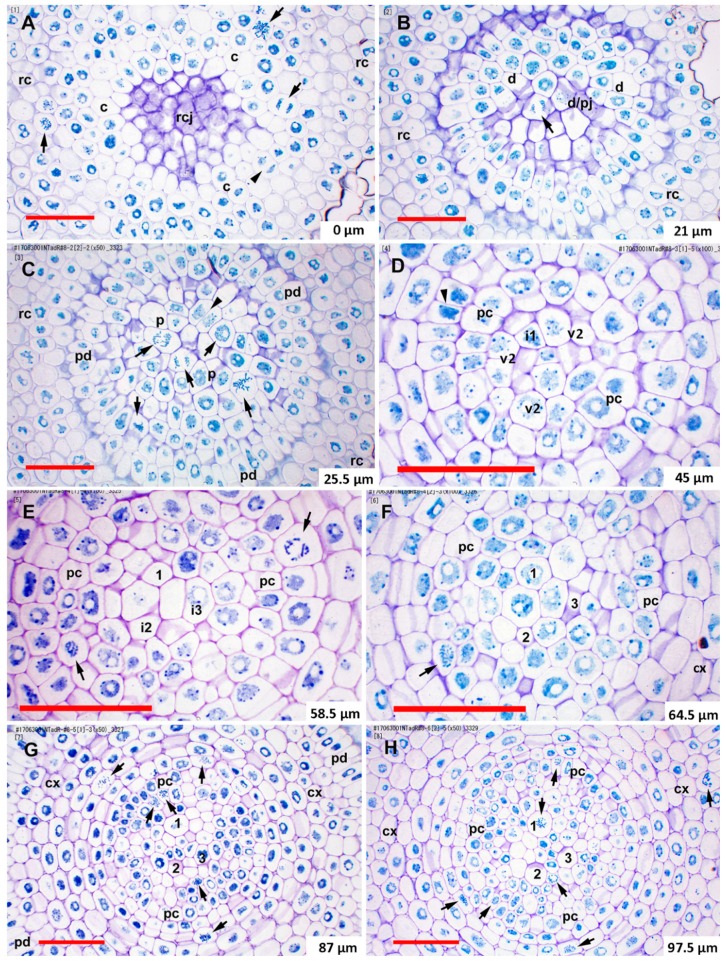
RNase-treated transverse sections featuring the histogens and procambium of a typical teosinte root tip taken during a rare period of mitotic activity in its quiescent center that also show the pattern of late-maturing metaxylem vessel (LMX) initiation and xylem tissue development. (**A**) The root cap junction (RCJ) cell walls are at center, acropetal view (toward the root cap); designated the 0 µm reference. Cells of the calyptrogen and root cap predominate. The acropetal end of a dermatogen-periblem central cell is visible at the center. (**B**). The dermatogen-perbilem complex/plerome junction (d/pj) 21 µm from the RCJ. The indicated mitotic cell is the plerome central cell. (**C**). Mitotically active plerome within an area normally reported to be mitotically “quiescent” 25.5 µm from RCJ. (**D**). Section through the 2nd vascular initials layer (v2) 45 µm from RCJ that includes the initial for the first LMX (i1). Transverse cell walls separating the two layers of vascular initials are present at center. (**E**). Section 58.5 µm from RCJ. Two more LMX initials are present (i2 and i3). The most acropetal cell of the first LMX file is at 1. (**F**). At 64.5 µm from RCJ three LMX files were differentiating (1-3 in order of initiation). (**G**). Cells were beginning to establish distinct collars around the LMX at 87 µm from RCJ. Parenchymatous cells were proliferating. (**H**). Encircling collars were established around all three LMX at 97.5 µm from RCJ. An LMX and other procambial cells can be seen in mitosis. Arrow, mitotic cell; arrowhead, newly forming cell plate; c, calyptrogen; cx, immature cortex; d. dermatogen-periblem complex; en, immature endodermis; i, LMX initial cell; p, plerome; pc, pericycle; pd, protoderm; rc, root cap; rcj, root cap/promeristem junction; v, vascular initials layer. Scale bar = 50 µm.

**Figure 3:**
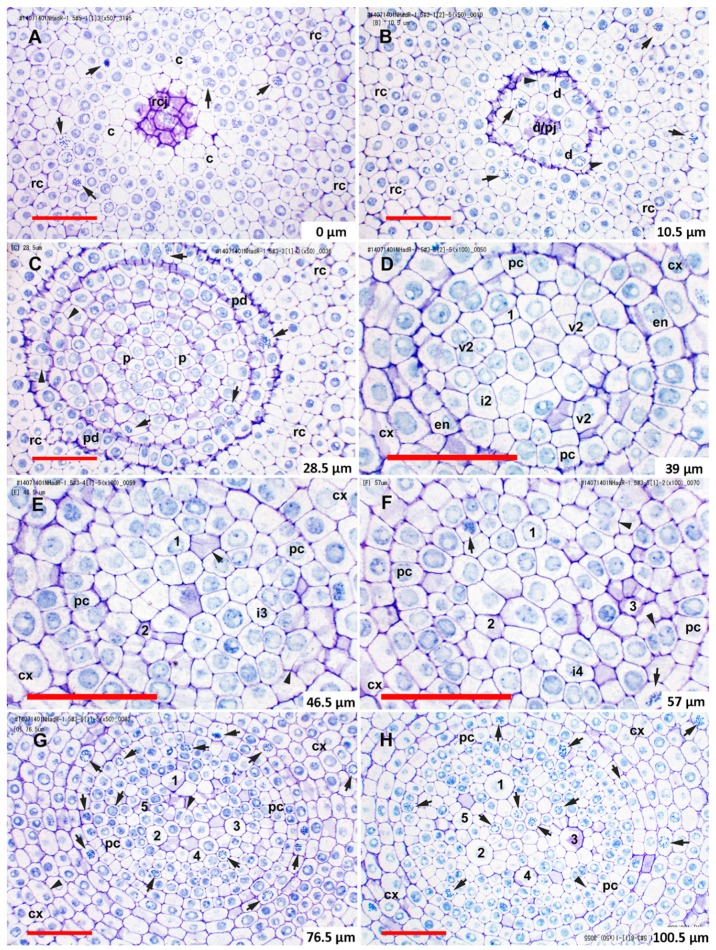
RNase-treated transverse sections featuring the histogens and procambium of a typical ‘Honey Bantam’ sweetcorn root tip showing the pattern of late-maturing metaxylem vessel (LMX) initiation and xylem tissue development. (**A**) The root cap junction (RCJ) cell walls are at center, acropetal view (toward the root cap); designated the 0 µm reference. Cells of the calyptrogen and root cap predominate. The acropetal end of a dermatogen-periblem central cell is visible at the center. (**B**) The dermatogen-perbilem complex/plerome junction (d/pj) 10.5 µm from the RCJ. (**C**) The plerome (within the quiescent center) 28.5 µm from RCJ. (**D**) Section through the 2nd vascular initials layer (v2) 39 µm from RCJ. Transverse cell walls separating two vascular initials layers are present at center and lower right. The most acropetal cell of the first LMX file is at 1 and the second LMX initial is at i2. (**E**) Section 46.5 µm from RCJ. Another LMX initial is present (i3). A pulse of mitotic activity had just finished. (**F**) At 57 µm from RCJ three LMX files were differentiating (1–3 in order of initiation). An LMX initial is present (i4). (**G**) Cells were beginning to establish distinct collars around the LMX at 76.5 µm from RCJ, and the most acropetal cell wall of the fifth LMX file is at 5. Numerous parenchymatous cells were proliferating in the procambium and immature cortex (ground meristem). (**H**) Encircling collars were established around the first four LMX at 100.5 µm from RCJ. Numerous parenchymatous cells were proliferating. Arrow, mitotic cell; arrowhead, newly forming cell plate; c, calyptrogen; cx, immature cortex; d, dermatogen-periblem complex; en, immature endodermis; i, LMX initial cell; p, plerome; pc, pericycle; pd, protoderm; rc, root cap; rcj, root cap/promeristem junction; v, vascular initials layer. Scale bar = 50 µm.

We found that treating tissue sections with RNase to remove cytoplasmic RNA before staining with toluidine blue O, which heavily stains cytoplasmic RNA, simplified evaluation of cell wall patterns and mitotic figure recognition by making cell walls and chromatin in nuclei more clearly visible (compare Figure 1 to Figure 2 and Figure 3). This enhanced our ability to use digital photography and computer-aided alignment to identify mitotic activity and very precisely track vessel element files and their immediate neighboring cells longitudinally. This was essential for accurately tracking a vessel to its apical terminus in serial transverse sections.

### 2.2. Histogen Organization in Teosinte and Sweetcorn

The root apical meristems of *Z. mays* ssp. *mexicana* teosinte had the closed anatomical organization typical of most grasses and were very similar to that of sweetcorn (Figure 1).

In most samples of teosinte and sweetcorn, based on staining density differences visible under standard toluidine blue staining and the general lack of mitotic figures and/or new cell walls, the quiescent centers (QC) were mainly limited to cells of the dermatogen-periblem complex, the plerome, and two somewhat less organized layers of cells basipetal to the plerome that contained procambial (vascular) initials and gave rise to all vascular cell types except pericycle. These four cell layers were centrally located at the tip of the root (Figure 1A,C), and we considered these to constitute the promeristem. Occasionally, histogen layers in particular roots stained more strongly (Figure 1D) or had significant mitotic activity (Figure 2C) despite their location in the putative QC.

The dermatogen-periblem and plerome were each usually organized as a distinct central cell or sometimes two cells surrounded by one (teosinte) or two-to-three (sweetcorn) rings of additional cells, and then the margin initials (Figure 1). There was similarity among promeristems of the two *Z. mays* types, which is noteworthy especially considering the significant differences in the ultimate dimensions of their steles (Figure 1, Figure 2 and Figure 3; Table 1 and Table 2). Immature cortex (ground meristem) and protoderm could be distinguished within one cell of a dermatogen-periblem margin by virtue of divisions periclinal to the planes of the histogens by the margin initials themselves or their first derivatives derived by anticlinal division. Subsequent periclinal divisions of the inner of those derivative cells initiated the differentiation of the endodermis from the rest of the ground meristem of the cortex (Figure 1A,C). In the procambium the pericycle cells were first to be differentiated, and these aligned directly with the cells of the plerome margin (Figure 1B). Accordingly, although mitosis in the plerome was only infrequently observed in general, the cell division plane was nearly always anticlinal with respect to the plane formed by the plerome cell layer. In other words, the plerome proliferated derivatives radially away from its margin, which was continuous with the pericycle (Figure 2C).

**Figure 4:**
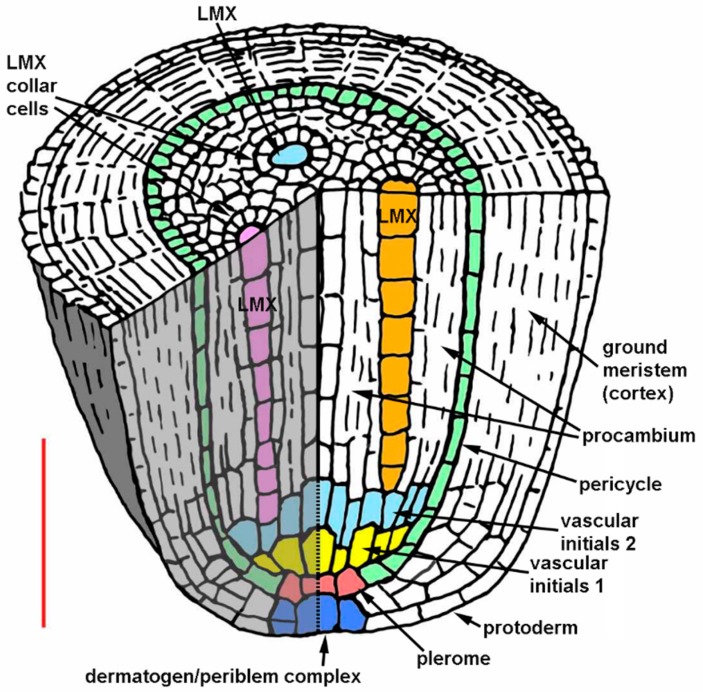
Schematic diagram of the promeristem and primary meristem of *Zea* primary roots. Highlighted with colors are the dermatogen-periblem complex, plerome, and vascular initials layers, the pericycle, and late-maturing metaxylem vessels (LMX) with their associated distinctive collar cells. The calyptrogen and root cap tissue are not shown. Scale bar = 100 µm.

The remainder of the procambium was produced by proliferation of cells from the two somewhat irregularly organized layers of vascular initial cells immediately basipetal to the plerome (Figure 1A,C). We distinguished this “vascular initials zone” from the plerome because it appeared to proliferate derivatives from its basipetal face and margin in a manner similar to the way the calyptrogen produced root cap cells, anticlinally at its margin and periclinally to the planes of the histogens from its basipetal face (Figure 1A and Figure 2C), and included the initials of the first-formed late-maturing metaxylem vessels (LMX; Figure 2D), though mitotic activity was rarely observed in this zone. Though they are the last to mature and become functional, the LMX elements were the second procambial cell type (after pericycle) to become recognizable, as previously reported [26,36]. Most primary roots of teosinte developed three to four large LMX, the first beginning within the vascular initials zone, typically 40–70 µm from the root cap junction depending on total histogen height (Figure 1 and Figure 2D–H, Table 1 and Table 2). Basipetal to 100µm from the RCJ these three to four LMX were joined by files of protoxylem, protophloem, and early-maturing metaxylem cells, but not additional LMX (data not shown). Sweetcorn initiated LMX in a similar manner (Figure 3D), but with significantly less variation in distance from the RCJ (Table 2), and ultimately produced significantly more LMX than teosinte within 100 µm from the RCJ (Figure 3D–H) and even more (ultimately six to seven) as maturation of primary tissue proceeded (data not shown). LMX usually began differentiating in a staggered fashion, though in teosinte with respect to distance from the RCJ the second LMX vessel typically became detectable at 7 µm or less compared to the position of the first and in sweetcorn it was a slightly greater span: 12 µm or less from the beginning of the first (Table 2). The later-initiating LMX became detectable in the procambium in no particular spatial orientation with respect to preexisting ones. Once LMX differentiation had begun, axially-oriented cell divisions in various planes occurred among the parenchymatous cells in the procambium until each LMX was usually surrounded by a distinct ring of cells of roughly the same size and shape (Figure 2G,H, Figure 3G,H; modeled in Figure 4).

**Figure 5:**
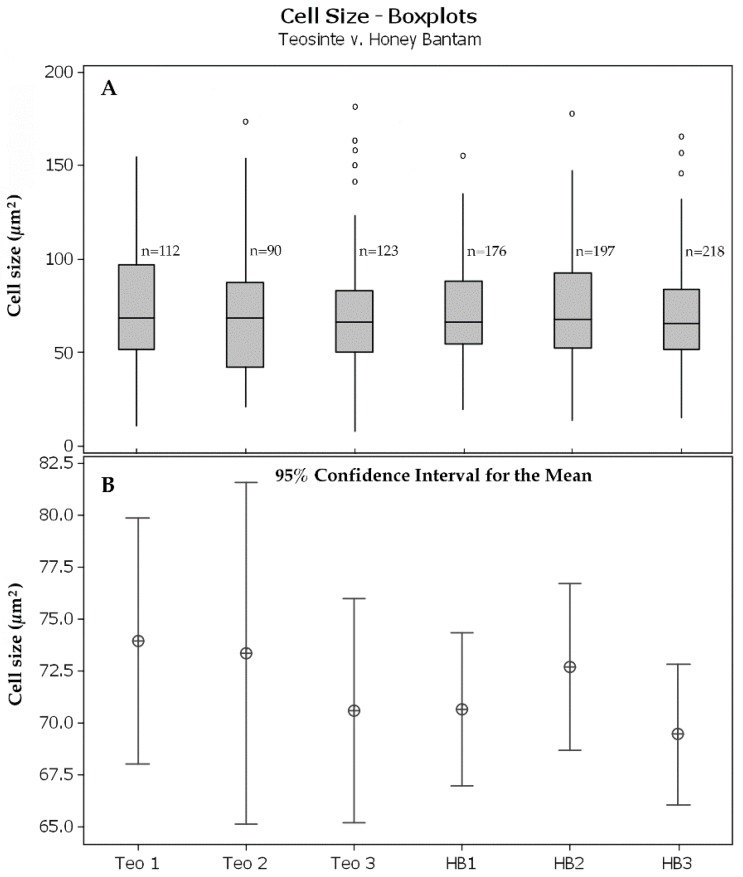
Analysis of transverse cytoplasmic areas of parenchymatous cells inside of the pericycle ca. 100 µm from the RCJ in teosinte and sweetcorn maize. (**A**) Boxplots of computed transverse cytoplasmic areas of teosinte and ‘Honey Bantam’ (*n* = 3 each). (**B**) 95% confidence intervals for mean parenchymatous cell areas from the same roots described in “A”. Mean cell areas were not significantly different (one-way ANOVA: F = 0.58, df = 5/890, *P* = 0.717).

At ca. 100 µm from the RCJ transverse areas of individual LMX of sweetcorn were slightly larger than those of teosinte, though the statistical significance of that result is weak likely due to the small number of LMX cells available in each root for measurement (Table 3). Average transverse areas of the numerous procambial parenchymatous cells inside the pericycles did not differ between teosinte and sweetcorn (compare Figure 2H to Figure 3H; Figure 5), which, along with cell count comparisons (teosinte 102 ± 28 vs. sweetcorn 197 ± 21; *t* = −4.72, df = 3.71, p = 0.011, *n* = 3), indicated that a larger number rather than a larger cell size of this cell type primarily accounted for the larger diameters of sweetcorn steles compared to those of teosinte (Table 1).

### 2.3. Mitotic Activity in the Promeristem

We defined “high mitotic activity” within a specific histogen layer as ≥ 5 cells in mitosis within the boundary of that histogen layer, as described above. With the exception of the calyptrogen, which typically displayed mitotic activity (Figure 1A, Figure 2A, Figure 3A), the histogens (including the vascular initials zone) that constituted the promeristem of most roots we observed were mitotically inactive except at their margins, consistent with the idea that histogens are centered in the QC. On the relatively rare occasions mitotic cells were observed in a histogen there were sometimes many (≥ 5) cells in mitosis at once within the boundaries of that histogen (Figure 2C). Two teosinte roots and two sweetcorn roots among ten transversely serially-sectioned roots of each type that were chosen at random and examined for mitotic activity in the QC had high levels of mitotic activity in one or more of their histogens.

## 3. Discussion

Morita and Nemoto [26] argued for a role by LMX in establishing the organization of the tissues of the crown root stele in rice because they begin differentiation before any other cell type except pericycle cells. Conversely, the classical view has been that the size of the QC and its influence on cell proliferation patterns are largely responsible for the positioning of the late metaxylem, particularly in *Sinapis alba* [24] and *Z. mays* (summarized by Feldman [6]). It was reported based on criteria used for analysis of barley vascular tissue by Heimsch [28], that in *Z. mays* four LMX elements begin differentiating simultaneously 40-80 µm from the RCJ [6,7]. The criteria specified by Heimsch [28] for identifying developing vessels using serial transverse sections from barley were two: “no change in the position of a member of a file in relation to cells surrounding it” and “no evidence of a recent longitudinal division in a file.” Feldman [6] added the word “radial” to the first criterion by specifying “radial position”. It was clear, however, from our transverse and longitudinal sections that LMX elements in *Zea mays* ssp. *mexicana* and in *Z. mays* cv. ‘Honey Bantam’, along with their surrounding cells, become displaced radially as the stele matures and that cells between and immediately surrounding the LMX occasionally undergo axially-oriented (i.e., longitudinal radial, oblique, and tangential) divisions that increase the number of cells in the vicinity of and between developing vessels (Figure 1A,C, compare Figure 2F–H and Figure 3F–H). This calls into question the utility of rigorously applying the first criterion described above for identifying differentiating xylem vessel files. Furthermore, LMX did not begin differentiating simultaneously (Table 2). Nevertheless, the first LMX file was distinctive in most cases to within one to two cells from the plerome, the region we refer to as the vascular initials layer (v1 and v2) and that we propose might therefore be considered a distinct histogen that could be called the “vasculogen”, and which performs in a manner analogous to the calyptrogen, except in the opposite direction, to produce the bulk of the procambium (Figure 4). We were able to consistently follow vessel files to their origins because our thin sectioning method consistently allowed us to identify the distal cell wall of the terminal cell in a developing vessel file, as was surely the case for Feldman [6]. We think the second criterion we cited above is valid because cells near the center of the first layer of vascular initials (v1), which is immediately basipetal to the plerome and in which the first LMX file initial could often be distinguished, mainly formed by anticlinal division with respect to the orientation of the histogens. When the plane of division could be ascertained (the longest of the mitotic phases, prophase, does not reveal division orientation), v1 cells usually divided in various axial planes (anticlinal), albeit rarely, but only transverse divisions (i.e., periclinal to the planes of the histogens) occurred in LMX files. No evidence was found that the plerome contributed to any other tissue but the uniseriate pericycle that radiated outward from the plerome’s margins. Interestingly, our teosinte data were in greater agreement with Feldman’s [6] ‘Kelvedon 33′ data than with our sweetcorn data were. We attributed the difference between measurement reported for ‘Kelvedon 33’ and ours for ‘Honey Bantam’ to cultivar differences, as was reported by Heimsch [28] for barley.

From an evolutionary perspective it is noteworthy that the organization of the promeristem, specifically the size and organization of the histogens in teosinte and sweetcorn (Figure 1, Table 1), were almost identical, as were typical cell sizes except for LMX (Figure 5; Table 3). This suggests that the main factor differentiating the two with regard to the development of the tissues of their steles, which were significantly broader quite soon in sweetcorn compared to teosinte, is the numbers of periclinal cell division events among parenchymatous cells of the procambium over developmental time. It further suggests that an increase of parenchymatous cell division frequency was positively selected naturally, or artificially by domestication, during the evolution of *Z. mays*.

Our most novel finding, however, was that on the relatively rare occasions that mitotic activity was found in the QC it was found with many mitotic figures in the same histogen layer (Figure 2C). In other words, our results suggest that the cell cycle may be very lengthy in cells of the QC, but when they occur mitoses are fairly tightly synchronized in a pulse. Twice daily periodicity in cell division activity has been reported in the RAMs of at least one species, *Allium cepa* [37,38], but the latter reported that mitotic events were distributed over 1–2 hr periods and did not occur in the position of the apical initials. Our observation was admittedly serendipitous, as it was not our intention to study cell-cycle behavior, and since all our root tips were arbitrarily fixed at noon, it is coincidental that our fixation time coincided with one of the peaks of activity reported by Kellicott [37] and Jensen and Kavaljian [38]. Despite this, coordinated mitoses in the promeristems of our roots was so infrequently observed, but striking when it occurred, that the synchrony is probably so tight in individual plants that it is frequently missed by conventional sampling practices. Given the report by Barlow and Macdonald [39] that the cell cycle time in cells of the QC is about 170 hr in *Z. mays* cv. ‘Golden Bantam’, it is understandable that mitoses would be frequently missed if they are synchronized, as we observed.

## 4. Conclusions

Metaxylem development in *mexicana* teosinte differed from sweetcorn only in that the numbers of late-maturing metaxylem vessels in the latter are typically two-fold greater and the number of cells in transverse section of procambium were greater in the latter, but parenchymatous cell sizes were the same. This suggests that over evolutionary time either the frequency of mitotic activity or its temporal persistence within the parenchymatous tissue of the procambium (or both) increased during domestication perhaps as a result of artificial selection. Perhaps this additional activity stimulated additional LMX initials to form. Verification of this hypothesis by future studies, especially at the molecular level, would be useful for comparing monocots with dicot systems in which such information has begun to be revealed. Primary root promeristems of both taxa were nearly identical in size and organization, but they did not operate quite as previously described. Mitotic activity was rare in the quiescent centers, as previously reported, but occasionally a synchronized pulse of mitoses was observed there. Our quantitative analysis and reinterpretation of histogen theory and procambium development should be useful for future detailed studies of the regulation of development, and perhaps its evolution, in this species.

## 5. Materials and Methods

### 5.1. Plant Material

Cultivation methods were modified after Gladish and Niki [40]. Teosinte seeds (*Zea mays* L. ssp. *mexicana* from Snow Brand Seed Co. Ltd., Sapporo, Japan) and sweetcorn (*Z. mays* cv. ‘Honey Bantam’ from Sakata Seed Corp., Yokohama, Japan) were surface sterilized in 10% (v/v) household bleach. 20–25 seeds each were sown under axenic conditions in 1 L beakers filled with moistened vermiculite (375 mL water/L vermiculite) that had been covered with foil and autoclaved. The beakers were then placed in constant 25 °C in a continuously dark growth chamber for 3 or 4 d (sweetcorn or teosinte, respectively). The 1 d difference was required because teosinte radicles typically required 1 d longer to emerge than those of sweetcorn. Consistently at noon one day after emergence, all primary roots that were 0.5–1.0 cm long were then collected for fixation and sectioning. The relative developmental time we chose for this study was early but was determined to be after the hiatus of cell division during radicle emergence and after the reestablishment of the QC [25].

### 5.2. Tissue Preparation for Light Microscopy (LM)

For conventionally prepared specimens, procedures for LM were modified from Niki et al. [41]. Root tip segments were excised from the selected roots and immediately immersed in 4% (w/v) paraformaldehyde in 0.1 M phosphate buffer and gently shaken overnight at room temperature. Following fixation, the specimens were rinsed in buffer, dehydrated by ethanol series, embedded in Technovit 7100™ resin (Heraeus Kulzer GmbH, Wehrheim, Germany), and serial sections were made transversely or longitudinally at 1.5 μm on a Reichert-Nissei UCT ultramicrotome (Leica Ltd., Tokyo, Japan). These were mounted on slides and stained with 0. 1% (w/v) pH 7 toluidine blue O (Electron Microscopy Sciences, Hatfield, PA, USA) at 45 °C for 2 min and rinsed with distilled water. Sections were viewed with a Leica DMLB light microscope (Leica Microsystems GmbH, Wetzlar, Germany) equipped with TU Plan Fluor ∞/0 lenses (Nikon, Tokyo, Japan) and photographed with a Canon Eos 5D Mark II digital camera (Canon Inc., Tokyo, Japan).

At neutral pH toluidine blue O principally stains carbohydrates and nucleic acids. Buffering the stain solution at pH 7.0 or lower prevents toluidine blue O from staining proteins in the cytosol to any significant degree. RNA is abundant in the cytosols of most meristematic cells, most of which lack vacuoles. Removing RNA by RNase treatment therefore makes nuclei, mitotic chromosomes, and cell walls more distinct after pH7 toluidine blue O staining by minimizing the staining of cytosolic contents. Therefore, some RNase-treated serial transverse and longitudinal sections were prepared for high resolution and high contrast LM micrographs as follows. Sections prepared as above in Technovit 7100™ resin and mounted on glass slides were treated with ribonuclease A (RNase; Sigma Chemical, St. Louis, MO, USA) at 20 µg/100 µL 0.05M phosphate buffer solution (pH 7.2). The slides were kept in an incubation box at 100% humidity at 37 °C for 4 h. The slides were then stained, viewed, and photographed as described above.

A total of 55 primary root tips of teosinte seedlings were serially sectioned transversely and 16 were serially sectioned longitudinally. A total of 19 seedling primary roots of the ‘Honey Bantam’ maize cultivar were serially sectioned transversely and 18 were serially sectioned longitudinally. All were examined microscopically after being stained by one of the two staining protocols. Of the transversely sectioned primary root tips of teosinte and sweetcorn seven of each type were selected at random for RNase treatment and subsequent detailed measurements. These constituted the physical “library” from which we sampled for the various comparisons reported here.

### 5.3. Cell Size Comparative Analysis

Digital photomicrographs of transverse sections cut 97.5–100.5 µm from the RCJ and prepared as described above and photographed at the same magnification from three roots each of teosinte and sweetcorn were prepared as follows for quantitation. With Photoshop CS6, all cells from and including the pericycle outwards were erased. The resulting images were “thresholded” at the same setting, and all cell contents that remained were erased, leaving only cell walls. The images were inverted, leaving only the cytoplasmic areas uniformly black. Vascular conducting cells (late-maturing metaxylem vessels [LMX] and the occasional protophloem sieve tube member) were identified from the original image and erased, which left only parenchymatous cells. With NIH Image 1.63, pixel sizes were calibrated to a photomicrograph of a stage micrometer taken at the same magnification, and the sectional areas of the cytoplasms were computed. Boxplots of area data for each section were generated, mean cell area 95% confidence intervals were computed and compared by one-way ANOVA, and mean vascular parenchymatous cell numbers and LMX cross-sectional area proportions of the procambium from three teosinte and three sweetcorn root transverse sections were compared using Student’s t-test with Minitab 16 (Minitab Inc., State College, PA, USA).

## Figures and Tables

**Table 1:** Structural data from primary root apical meristems (promeristems) of teosinte (*Zea mays* ssp. *mexicana*) and sweetcorn (*Z. mays* ‘Honey Bantam’) 3–4 d after germination in moist vermiculite (means ± std. dev., n = 7 each). Stele diameters are shown at the plerome and at fixed distances from the root cap junction. All teosinte stele diameters were significantly different (*t*-test, *p* = 0.05) than sweetcorn. Histogen heights were not significantly different (*t*-test, *p* = 0.05) between the taxa (statistics comparing column values are below each column).

Taxon	Histogen Heights (μm) at Center				Stele Diameters (μm)		
	Dermatogen/Periblem	Plerome	First Vascular Initials Layer	Total ht	Plerome	@60μm	@100μm
Teosinte	15.9 ± 5.7	11.6 ± 3.9	12.1 ± 6.5	39.6 ± 15.1	56.0 ± 6.5	99.0 ± 5.7	129.9 ± 5.8
Sweetcorn	14.1 ± 2.4	9.4 ± 2.5	9.2 ± 2.9	32.7 ± 4.8	65.7 ± 6.1	130.0 ± 15.2	182.4 ± 9.7
*t*	0.77	1.27	1.08	1.16	−2.90	−5.14	−12.29
df	8	10	8	7	11	7	9
*P*	0.464	0.234	0.311	0.285	0.014	0.001	0.000

**Table 2:** Late-maturing metaxylem vessel (LMX) initiation data. Indicated are initiation distance of the first three LMX cell files measured from the root cap junction and distances from the basipetal face of the first layer of vascular initials (vascular initials1) to the most acropetal LMX cell and the distance between the initiation of the first and second LMX cell file. (Means ± std. dev., *n* = 7 each.) Measurements were not significantly different (*t*-test, p = 0.05) between the taxa threshold due largely to high variation in teosinte. Statistics comparing column values are below each column.

Taxon	Initiation of LMX Cell Files (µm)			Vascular Initials1 to 1st LMX (μm)	Distance Between 1st and 2nd LMX Initiation (μm)
	First	Second	Third		
Teosinte	50.1 ± 19.7	54.5 ± 21.2	59.9 ± 19.7	10.4 ± 6.3	4.4 ± 2.6
Sweetcorn	37.9 ± 3.8	45.4 ± 4.1	48.2 ± 5.5	5.1 ± 4.7	5.1 ± 4.7
*t*	1.611	1.110	1.510	−1.77	−1.56
df	6	6	7	11	9
*P*	0.155	0.307	0.175	0.105	0.154

**Table 3:** Mean (± std. dev.) LMX cell cross-sectional area (µm^2^) at approximately 100 µm from the RCJ. The difference between taxa was not statistically significant.

Teosinte			‘Honey Bantam’ Sweetcorn		
Teo 1	Teo 2	Teo 3	HB 1	HB 2	HB 3
225.4 ± 56.0	194.6 ± 6.0	239 ± 46.7	268.3 ± 75.6	325.5 ± 46.0	318.1 ± 101.8
*n* = 3	*n* = 3	*n* = 4	*n* = 5	*n* = 5	*n* = 6

ANOVA: F = 2.50; df 5/21; *P* = 0.063.

## Abbreviations

LMX	late-maturing metaxylem vessel
QC	quiescent center
RCJ	root cap junction
RFLP	restriction fragment length polymorphism
RAM	root apical and primary meristem zones
